# Clinical efficacy of moluodan in the treatment of chronic atrophic gastritis: A protocol for systematic review and meta-analysis

**DOI:** 10.1097/MD.0000000000032303

**Published:** 2022-12-30

**Authors:** Yunfeng Yu, Xinyu Yang, Gang Hu, Shuang Yin, Fei Zhang, Yandong Wen, Ying Zhu, Zhenjie Liu

**Affiliations:** a The First Affiliated Hospital of Hunan University of Chinese Medicine, Changsha, Hunan, China; b Hunan University of Chinese Medicine, Changsha, Hunan, China; c Xiyuan Hospital of China Academy of Chinese Medical Sciences, Beijing, China.

**Keywords:** chronic atrophic gastritis, clinical efficacy, meta-analysis, moluodan, trial sequential analysis

## Abstract

Chronic atrophic gastritis (CAG) is an important stage of precancerous lesions of gastric cancer, and also a key period of drug intervention. However, there is still a lack of drugs to maintain the treatment of CAG until the advent of moluodan.

**Objective:**

This study was conducted to assess the clinical efficacy of moluodan in the treatment of CAG by meta-analysis and trial sequential analysis.

**Methods:**

China National Knowledge Infrastructure, China Biology Medicine, VIP, Wanfang, Embase, PubMed, the Cochrane Library, and Web of Science databases were searched, all with the time limit from database establishment to July 2022. The published randomized controlled trials of moluodan for CAG were conducted for meta-analysis and trial sequential analysis.

**Results:**

7 studies with a total sample size of 1143 cases were included. Compared to folic acid/vitamins, moluodan alone significantly increased the effective rate of pathological detection (relative risk [*RR*] = 1.73, 95% confidence interval [95%*CI*] = [1.48,2.02], *P* < .00001), and moluodan in combination with folic acid/vitamins significantly increased the effective rates of pathological detection (*RR* = 1.37, 95%*CI* = [1.23,1.52], *P* < .00001), gastroscopy (*RR* = 1.37, 95%*CI* = [1.18,1.60], *P* < .0001) and symptoms (*RR* = 1.25, 95%*CI* = [1.13,1.38], *P* < .0001). Harbord regression showed no publication bias (*P* = .22). Quality of evidence evaluation demonstrated moderate quality of evidence for all indicators.

**Conclusions:**

Moluodan can improve the effective rates of pathological examination, gastroscopy and symptoms in patients with CAG, and play a role in slowing down the disease progression and reducing clinical symptoms. It may be a potential drug for the treatment of CAG and has the value of further exploration.

## 1. Introduction

Chronic atrophic gastritis (CAG) is a chronic inflammatory disease of the gastric mucosal tissue characterized by atrophy of the gastric mucosal glands, which may be accompanied by intestinal metaplasia and dysplasia.^[1]^ Available studies suggest that “chronic non-atrophic gastritis - chronic atrophic gastritis - intestinal epithelial hyperplasia - atypical hyperplasia” is the unique progression pattern of gastric cancer.^[2]^ CAG is a precancerous lesion of gastric cancer,^[3]^ and its development is associated with helicobacter pylori (HP) infection.^[4]^ It has been shown that eradication of HP can slow down the progression of gastric precancerous lesions^[5,6]^ and reduce the incidence of gastric cancer in infected populations.^[7]^ However, some patients with CAG continue to experience persistent discomfort after eradication of HP, and these symptoms significantly affect the patient’s daily life.^[8]^ Additionally, the incidence of gastric cancer in patients with severe CAG can still reach 116/1,00,000 people after eradication of HP,^[9]^ seriously endangering patients’ lives and health. Therefore, patients with CAG still need maintenance therapy after eradication of HP to further reduce the risk of gastric cancer and improve clinical symptoms. The Chinese Integrated Guideline on Clinical Management of Gastric Precancerous Conditions and Lesions state that folic acid and antioxidant vitamins are effective in improving the prognosis of CAG,^[10]^ but the Management of Epithelial Precancerous Conditions and Lesions in the Stomach (MAPS II) do not recognize their benefit,^[2]^ and CAG still lacks effective drugs for maintenance therapy. Therefore, it is necessary to explore other treatment options that can improve the prognosis of CAG.^[7]^

Moluodan is a compound prescription of Chinese herbal medicine which developed by Professor Li EF, consisting of baizhu (Atractylodes Macrocephala), fuling (Poria), jineijin (Endothelium Corneum Gigeriae Galli), jiujiechangpu (Anemone altaica Fisch), zexie (Alismatis Rhizoma), puhuang (Pollen Typhae), sanqi (Notoginseng Radix), chuanxiong (Chuanxiong Rhizoma), danggui (Angelicae Sinensis Radix), diyu (Sanguisorbae Radix), baihe (Lilii Bulbus), shihu (Dendrobii Caulis), maidong (Ophiopogon japonicus (Linn. f.) Ker-Gawl), xuanshen (Scrophulariae Radix), yanhusuo (Corydalis Rhizoma), wuyao (Linderae Radix), baishao (Paeoniae Radix Alba).^[11]^ Moluodan has been included in the MAPS II^[2]^ in 2019 and recommended as a treatment for gastric precancerous lesions, especially mild dysplasia, in the Guidelines for the Clinical Application of Traditional Chinese Medicine in the Treatment of Chronic Gastritis^[12]^ in 2020. The present study suggests that moluodan may increase serum gastrin by repairing gastrin-secreting cells and somatostatin-secreting cells in the gastric mucosa to treat CAG.^[13]^ In addition, moluodan also significantly reduced the expression of EGF and EGFR in the serum of patients with CAG, thereby inhibiting further transformation of precancerous lesions in gastric cancer.^[14]^ In recent years, an increasing number of clinical trials have suggested that moluodan has good efficacy in the treatment of CAG, but there are no relevant systematic evaluations and meta-analysis. Thus, this study used meta-analysis and trial sequential analysis (TSA) to evaluate the clinical efficacy of moluodan in the treatment of CAG, aiming to provide an evidence-based justification for the clinical application of moluodan.

## 2. Methods

This study strictly followed the preferred reporting items for systematic reviews and meta-analyses.^[15]^

### 2.1. Literature Search

The databases of China national knowledge infrastructure, China biology medicine, VIP, Wanfang, Embase, PubMed, the Cochrane Library, and Web of Science were searched for clinical studies of moluodan in the treatment of chronic atrophic gastritis, all with the time limit from database establishment to July 2022. English subject terms covered moluodan, chronic atrophic gastritis, and Chinese subject terms covered moluodan, chronic atrophic gastritis. On the basis of the subject terms, we expanded the Chinese free terms by using China national knowledge infrastructure and China biology medicine database, and the English free terms by using MeSH database and the Cochrane Library, and then combined the subject terms and free terms for searching.

### 2.2. Inclusion and exclusion criteria

The inclusion criteria are listed below: Study design: Randomized controlled trials; Participants: Conforms to the basic diagnosis of chronic atrophic gastritis;^[16]^ Intervention: Patients in the control group were given folic acid and/or vitamins and patients in the experimental group were given moluodan or moluodan combined with the control regimen; Outcomes: Effective rate of pathological examination was used as the primary efficacy endpoint, effective rates of gastroscopy and symptoms were used as secondary efficacy endpoints, and total adverse events were used as safety endpoints. (The effective rate of pathological examination refers to the reduction of acute and chronic inflammation by more than 1 degree, and the reduction of glandular atrophy, enterosis and dysplasia. The effective rate of gastroscopy refers to the reduction of mucosal lesions by more than 1/2 degree and the reduction of inflammation by gastroscopy. The effective rate of symptoms refers to significant reduction of major symptoms.)

The exclusion criteria are as follows: Studies such as reviews, animal experiments, and case reports; Repeatedly published studies; Studies published in abstract form; Studies with incomplete or unclear data.

### 2.3. Literature screening, data statistics and risk of bias

Firstly, the base literature retrieved from each database was imported into Endnote X9. After reading the title and abstract, and reviewing the full text, the literature was eliminated in order based on the inclusion criteria, and the included literature was finally identified. Secondly, the included literature was classified and organized to extract basic characteristics such as author, year, sample size, mean age, sex ratio, intervention, and course of treatment and entered into the data statistical table. Finally, the Cochrane risk of bias assessment tool was used to assess the risk of bias according to the requested entries. All the above work was carried out independently by 2 investigators, and any disagreement was adjudicated by a third investigator.

### 2.4. Statistical analysis

Meta-analysis was conducted using Revman5.3. Relative risk (RR) and 95% confidence interval (95% CI) were used as effect sizes for dichotomous variables. Continuous variables used mean difference and 95% CI as effect sizes. Heterogeneity was analyzed by *I*^*2*^ test and *Q* test. If *I*^*2*^ < 50% and *P* > .1, the heterogeneity was small and fixed-effect model analysis was used. Otherwise, random effects model analysis was used. In case of indicators with significant heterogeneity, sensitivity analysis would be performed. That is, after excluding 1 study at a time, the remaining studies were combined for the analysis. The results were shown to be robust if there was no significant change in the continuous variables obtained from each combined analysis.

TSA0.9.5.10 Beta software was utilized to perform the TSA. The original results are conclusive if the cumulative Z value crosses the desired information value or TSA bound. Stata15.0 software was evaluated for publication bias, and if Harbord regression indicated *P* > .1, there was no publication bias. GRADEpro3.6 software was applied to evaluate the quality of evidence, and the evaluation method was referred to the GRADE evidence evaluation guidelines.

## 3. Results

### 3.1. Result of literature search

**Figure 1.**
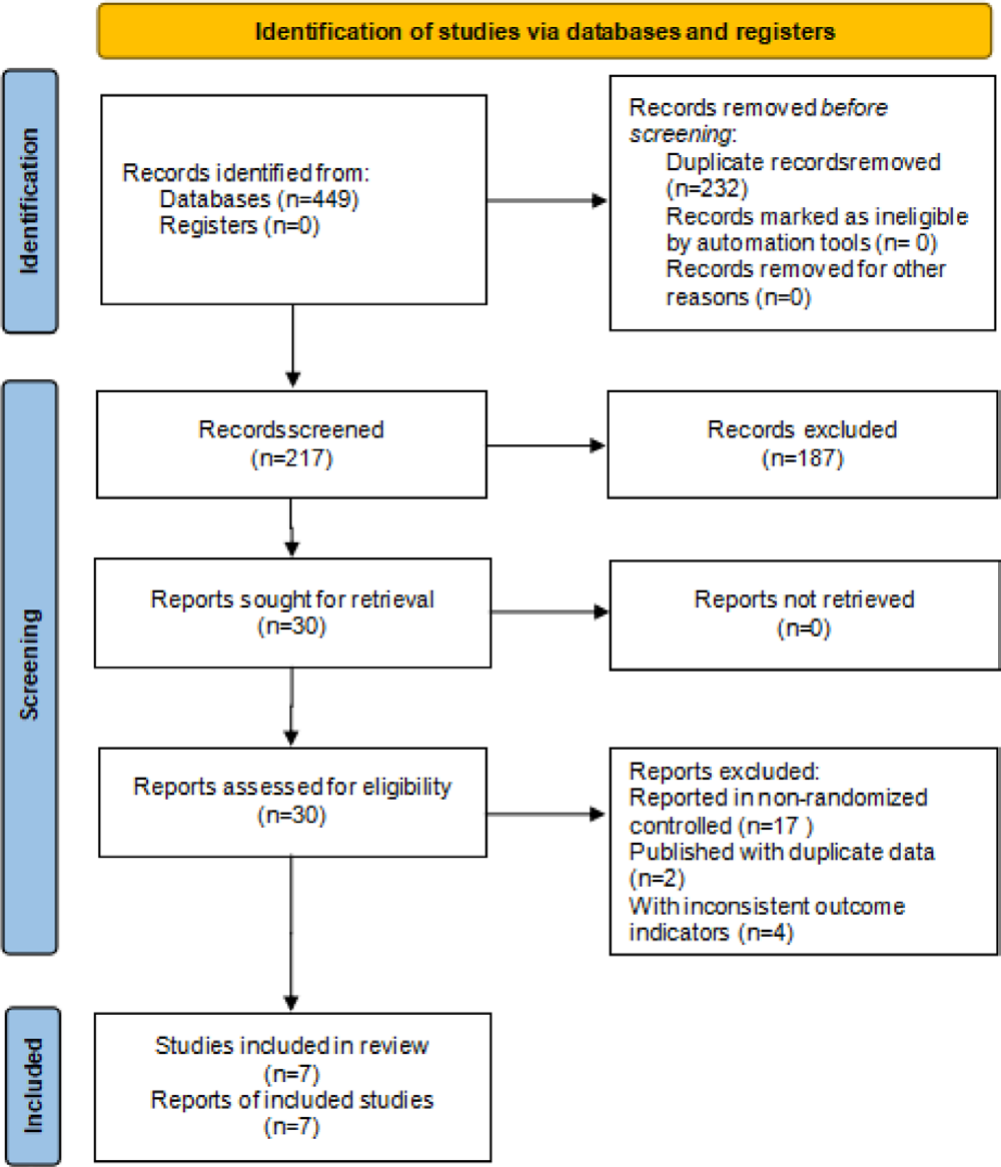
The flow chart of literature screening.

A total of 449 literatures were retrieved, 232 duplicates were excluded, 187 literatures were filtered out after reading the titles and abstracts, 23 literatures were removed after reviewing the full text, and 7 literatures were finally included.^[17–23]^ As shown in Figure 1.

### 3.2. Basic Materials

A total of 7 clinical studies were included, with a total sample size of 1143 cases, 603 in the experimental group and 540 in the control group. The research centers were all located in China. Two studies with 592 cases reported “moluodan versus folic acid/vitamins” and 5 studies with 551 cases reported “moluodan + folic acid/vitamins versus folic acid/vitamins.” Of the included studies, only Zhang MM 2016^[21]^ reported adverse events and therefore meta-analysis of safety endpoints could not be performed. The basic characteristics of the included studies are shown in Table 1.

**Table 1:** Basic characteristics of the included studies.

ID	age/yrs	course of disease/yrs	male/female	sample size			experience arm	control arm	course of treatment/mo
				Experience	control	Pathology			
Tang XD 2015	58.79	/	214/182	200	196	Mild to moderate dysplasia	Moluodan 9g tid Placebo 5mg tid	Folate 5mg tid Placebo 9 g tid	6
Du AM 2015	60.00	/	133/63	130	66	/	Moluodan 8pills tid	Folate 10 mg tid Vitamin E 10mg tid	6
Feng RB 2011	43.51	15.04	63/42	54	51	Mild to moderate dysplasia	Moluodan 1piece tid Folate 10mg tid	Folate 10mg tid Placebo 1piece tid	3
Li G 2012	41.50	15.15	103/65	84	84	Mild to moderate dysplasia	Moluodan 8pills tid Vitamin B_12_ 50ug q2d	Vitamin B_12_ 50 ug q2d	3
Zhang MM 2016	34.52	6.05	29/31	30	30	/	Moluodan 8pills tid Vitacoenzyme 0.8g tid	Vitacoenzyme 0.8 g tid	3
He DX 2017	44.50	12.75	67/53	60	60	LGIN	Moluodan 5pills tid Vitamin E 10mg tid	Vitamin E 10 mg tid	6
Shi YM 2017	53.53	6.17	60/49	55	54	/	Moluodan 8pills tid Folate 10mg tid	Folate 10 mg tid	3

### 3.3. Risk of bias assessment

**Figure 2.**
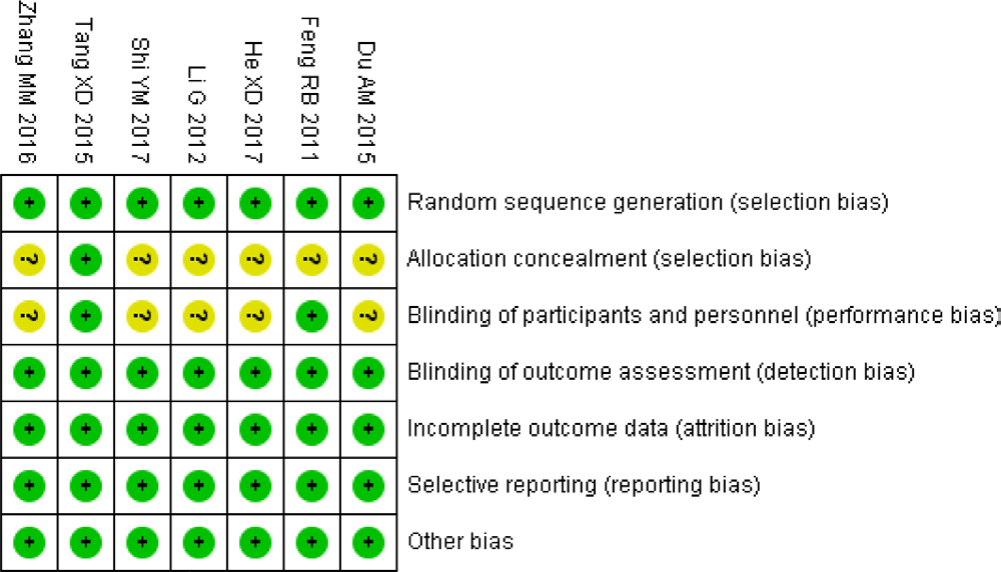
Risk of bias assessment diagram.

Of the 7 included studies, the risk of hidden allocation in 6 and intervention-blinding to patients in 5 were unclear, and the risk of bias in the remaining domains was low. The risk of bias for the included studies is shown in Figure 2.

### 3.4. Efficacy endpoints

#### 3.4.1. Moluodan versus Folic acid/vitamins.

**Figure 3.**
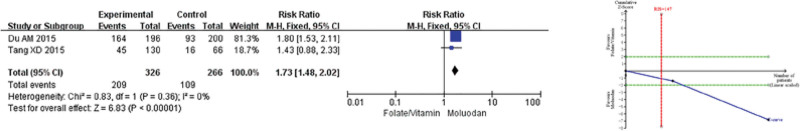
Meta-analysis and TSA of efficacy endpoints of “moluodan versus folic acid/vitamins.” TSA = trial sequential analysis.

Two studies were included in the comparison of “moluodan versus folic acid/vitamins.” Meta-analysis results revealed a significantly higher effective rate of pathological examination (*RR* = 1.73, 95%*CI* = [1.48,2.02], *P* < .00001) in the moluodan group compared to the folic acid/vitamin group. The TSA demonstrated conclusive results observed for the current information size and GRADE showed moderate quality of evidence for this indicator (Fig. 3).

#### 3.4.2. Moluodan + Folic acid/vitamins versus Folic acid/vitamins.

**Figure 4.**
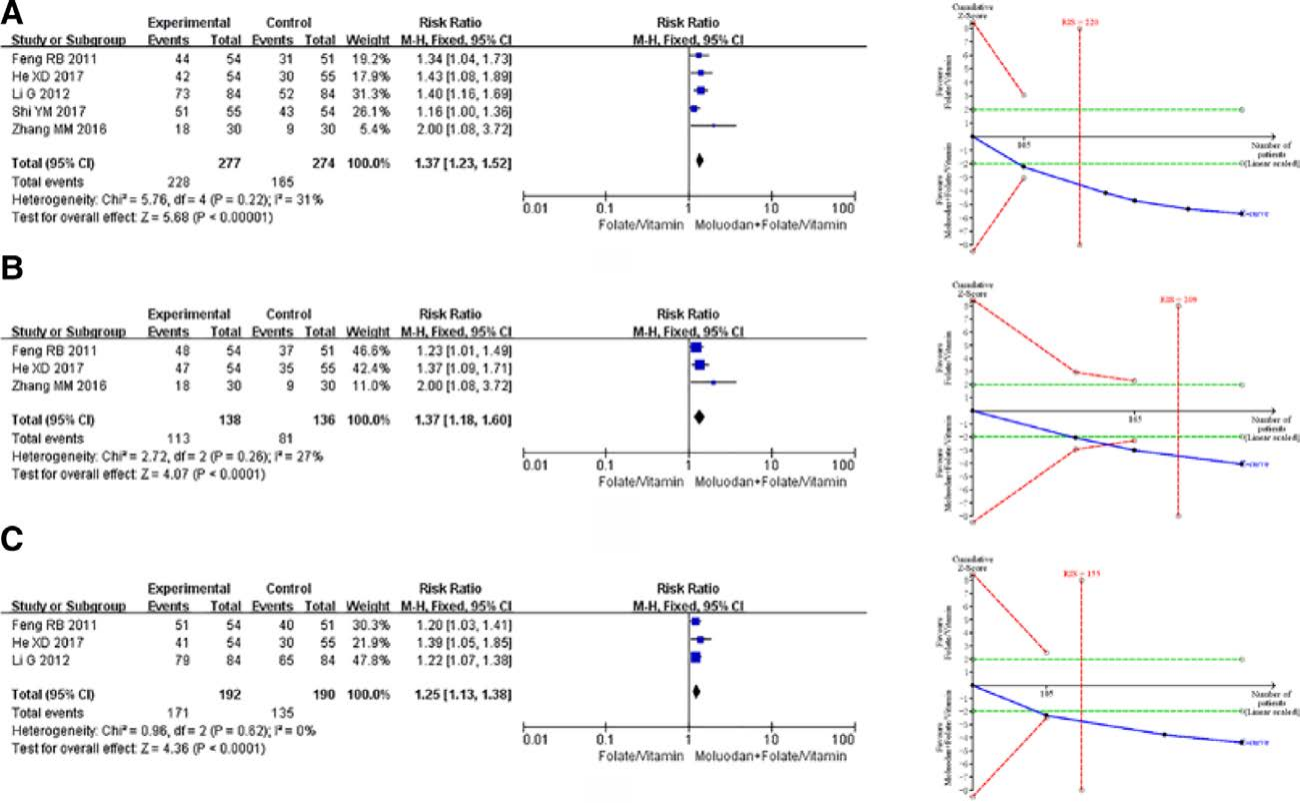
Meta-analysis and TSA of efficacy endpoints of “moluodan + folic acid/vitamins versus folic acid/vitamins.” TSA = trial sequential analysis.

Five studies were included in the comparison of “moluodan + folic acid/vitamins versus folic acid/vitamins.” Meta-analysis showed that compared with the folic acid/vitamin group, the moluodan + folic acid/vitamin group had significantly higher effective rates of pathological examination (*RR* = 1.37, 95%*CI* = [1.23,1.52], *P* < .00001), gastroscopy (*RR* = 1.37, 95%*CI* = [1.18,1.60], *P* < .0001), and symptoms (*RR* = 1.25, 95%*CI* = [1.13,1.38], *P* < .0001). The TSA suggested that the results observed for the current amount of information were conclusive, and GRADE indicated a moderate quality of evidence for these indicators (Fig. 4).

### 3.5. Subgroup analysis

Subgroup analysis was performed using the effective rate of pathological examination as an evaluation index, with the drug and the course of treatment as the subjects respectively. Subgroup analysis of the drugs indicated that moluodan in combination with folic acid significantly increased the effective rate of pathological examination by about 24% compared to folic acid, and moluodan in combination with vitamins significantly increased the effective rate of pathological examination by about 47% compared to vitamins. Subgroup analysis of the course of treatment showed that compared to the folic acid/vitamin group, the moluodan combination group improved the effective rate of pathological examination by approximately 35% after 3 months of treatment and by approximately 43% after 6 months of treatment. Subgroup analysis of pathology showed that compared to the folic acid/vitamin group, the moluodan combination group significantly increased the effective rate of pathological examination by approximately 43% for CAG with low-grade intraepithelial neoplasia and by approximately 38% for CAG with mild to moderate dysplasia. As shown in Table 2.

**Table 2:** Subgroup analysis of drugs, courses and pathology.

Factor	Subgroup	*I* ^*2*^	RR(95%CI)	*P* value
Drug	Folate	0	1.24(1.08.1.43)	.003
	Vitamin	0	1.47(1.26,1.72)	<.00001
Course of treatment	3mo	44	1.35(1.20,1.52)	<.00001
	6mo	0	1.43(1.08,1.89)	.01
Pathology	LGIN	0	1.43(1.08,1.89)	.01
	Mild to moderate dysplasia	0	1.38(1.19,1.60)	<.0001

### 3.6. Publication bias assessment

**Figure 5.**
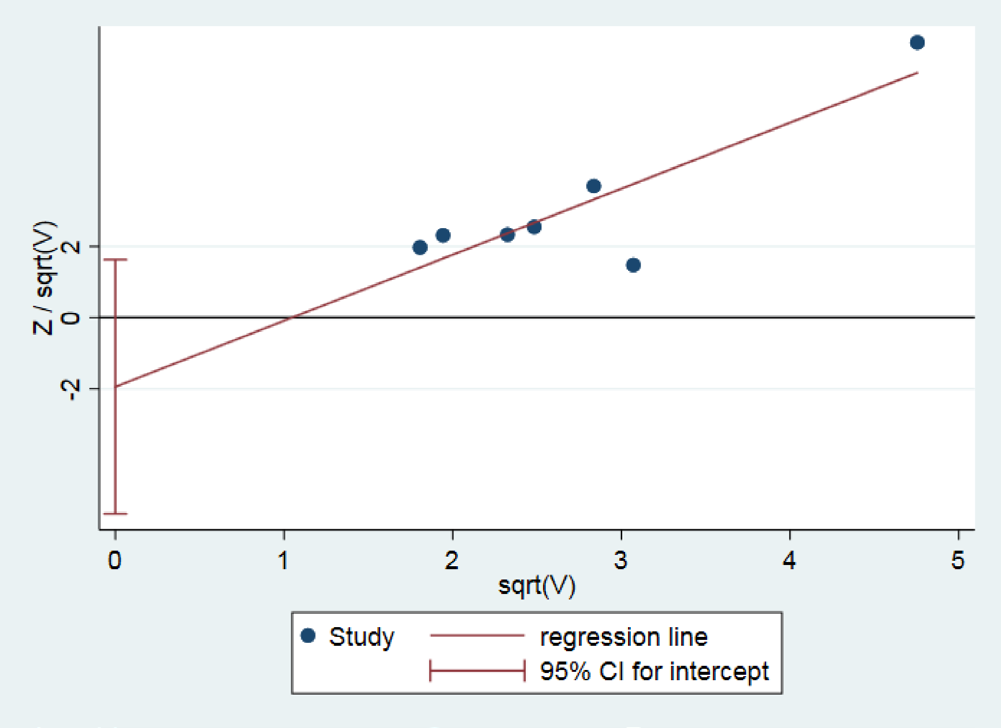
Harbord regression of the effective rate of pathological examination.

Harbord regression of effective rate of pathological examination showed no significant publication bias (*P* = .22) (Fig. 5).

## 4. Discussion

A total of 7 clinical trials and 1143 sample sizes were included in this study, which systematically evaluated the efficacy of moluodan alone or in combination with folic acid/vitamins for the treatment of CAG. It was the 1^st^ meta-analysis and TSA of moluodan on CAG so far. The TSA and quality of evidence evaluation gave a more comprehensive and credible result for this study.

CAG is a chronic inflammation of the gastric mucosa caused by various reasons, in which upper abdominal pain, fullness, acid reflux, belching and loss of appetite are the main clinical manifestations.^[16]^ CAG is often seen in conjunction with atypical hyperplasia^[24]^ and there is evidence that severe atypical hyperplasia may lead to a significant increase in the rate of cancer in this disease,^[25]^ seriously endangering the life and health of patients. Currently, early discovery and diagnosis of CAG as well as early treatment are effective measures to prevent gastric cancer.^[26]^ White light endoscopy and endoscopic biopsy are considered to be the most common diagnostic and follow-up methods for CAG.^[27]^ Therefore, in this study, the effective rate of pathological examination was used as the primary efficacy endpoint, and the effective rates of gastroscopy and symptoms were used as secondary efficacy endpoints.

According to Chinese medicine, GAC belongs to the category of “weiwantong, and piman,”^[28]^ and the basic pathogenesis is the weakness of the spleen and stomach with stasis of blood,^[29]^ so Chinese medicine treatment should be based on the principle of strengthening the spleen and invigorating blood circulation. Moluodan is a compound prescription of pure Chinese herbal medicine, covering a total of 18 herbs. It has the function of strengthening the spleen and dispelling dampness, activating blood circulation and resolving stasis, harmonizing the stomach and relieving pain, and its composition fits the Chinese medical mechanism of CAG.

Meta-analysis showed significantly higher effective rates of pathological examination in the moluodan group compared to folic acid or vitamins, and TSA indicated that the current results were conclusive, suggesting that moluodan is effective in slowing the pathological progression of CAG. Du et al^[18]^ also reported that moluodan was able to relieve clinical symptoms such as epigastric distention, belching, heartburn, and anorexia. Compared with folic acid or vitamins, the moluodan combination group showed significantly higher effective rates of pathological examination, gastroscopy and symptoms by 37%, 37% and 25%, and TSA demonstrated that these results were conclusive. This suggested that the combination of moluodan was effective in alleviating the CAG process and improving the symptoms of patients. Subgroup analysis revealed that moluodan combined with folic acid increased the effective rate of pathological detection by about 24% compared to folic acid alone, and moluodan combined with vitamins increased the effective rate of pathological detection by about 47% compared to vitamins alone. This implies that moluodan can exert its therapeutic effect either in combination with folic acid or in combination with vitamins. In addition, compared to the folic acid or vitamin group, the moluodan combination group increased the effective rates of pathological examination by 35% and 43% after 3 and 6 months of treatment, respectively. This implies that moluodan achieves benefit in all 3 to 6 months and that the efficacy of treatment may improve with the increase of treatment course.

Subgroup analysis revealed that the moluoldan combination group increased the effective rates of pathological examination by 43% and 38% for low-grade intraepithelial neoplasia and mild to moderate dysplasia, respectively, when compared with folic acid/vitamins. This confirms the role of moluodan in improving the prognosis of low-grade intraepithelial neoplasia and mild-to-moderate dysplasia. This is in line with the opinion of the Guidelines for clinical application of traditional Chinese medicine in the treatment of chronic gastritis (2020). Subgroup analysis could not be localized to intestinalization, severe dysplasia, and intermediate and high-grade intraepithelial neoplasia. This is because some of the included studies covered patients with both intestinalization, dysplasia, and intraepithelial neoplasia, and some studies subjectively excluded patients with severe dysplasia.In terms of safety, Zhang MM et al^[21]^ found that the adverse events of moluodan combined with vitacoenzyme tablets were comparable to those of vitacoenzyme tablets, suggesting that moluodan may have a good safety profile. Meta-analysis of safety endpoints has not been performed due to the limitation of the literature base.

The quality of evidence evaluation demonstrated that the quality of evidence for all indicators was moderate, suggesting that this study has some limitations (shown below): The narrow total sample size reduces the confidence of the analysis results. Although widening the inclusion criteria may increase the base and total sample size of the study, this may also raise the risk of bias; There was some bias in the design of the study. Six of the included studies have an unclear risk of hidden allocation and 5 have an unclear risk of intervention-blinding to patients, which may lead to an increased risk of selection bias and implementation bias; The narrow inclusion criteria may have reduced the generalizability of the results. Tang XD et al^[17]^ restricted the age criterion to < 70 years, and He XD et al subjectively excluded patients with dysplasia, and these restrictions may reduce the generalizability of the results to some extent; The follow-up period is short. The current treatment of CAG is a long-term process, so the long-term efficacy of moluodan for CAG is more important. Among the included studies, only He XD,^[22]^ Du AM,^[18]^ and Tang XD^[17]^ had a 6-month course of treatment, while the rest^[19–21,23]^ had a 3-month course, which means that the current study mainly reflects the medium-term efficacy of moluodan for CAG, and its long-term efficacy still needs to be verified in future studies.

## 5. Conclusion

Moluodan can improve the effective rates of pathological examination, gastroscopy and symptoms in patients with CAG, and play a role in slowing down the progression of disease and alleviating clinical symptoms, which may be a potential drug for the treatment of CAG and has the value of further exploration.

## Acknowledgments

We would like to thank Gaowen Wei for providing statistical guidance.

## Author contributions

**Conceptualization:** Yu YF.

**Data curation:** Yang XY.

**Formal analysis:** Yu YF, Yang XY.

**Investigation:** Hu G, Yin S.

**Methodology:** Hu G.

**Software:** Zhang F, Yin S.

**Supervision:** Wen YD.

**Writing – original draft:** Yu YF, Yang XY.

**Writing – review & editing:** Zhu Y, Liu ZJ.
